# Craniofacial characteristics and cosmetic satisfaction of patients with sagittal and metopic synostosis: a case–control study using 3D photogrammetric imaging

**DOI:** 10.1007/s00381-021-05434-y

**Published:** 2021-12-23

**Authors:** Anja Svalina, Ville Vuollo, Willy Serlo, Juha-Jaakko Sinikumpu, Anna-Sofia Silvola, Niina Salokorpi

**Affiliations:** 1 Department of Neurosurgery, Oulu University Hospital, Oulu, Finland; 2 Medical Research Center, Oulu University Hospital, Oulu, Finland; 3 Faculty of Medicine, Research Unit of Oral Health Sciences, University of Oulu, Oulu, Finland; 4 Department of Children and Adolescent, Oulu University Hospital, Oulu, Finland; 5 PEDEGO Research Group, University of Oulu, Oulu, Finland; 6 Department of Orthodontics, Faculty of Medicine, Oral Health Sciences, University of Oulu, Oulu, Finland; 7 Research Unit of Clinical Neuroscience, Oulu University Hospital and University of Oulu, Oulu, Finland

**Keywords:** Craniosynostosis, Aesthetical outcome, Craniofacial symmetry, Metopic, Sagittal, Adult

## Abstract

**Purpose:**

The aim of this study was to investigate the craniofacial and aesthetic characteristics of adult metopic and sagittal craniosynostosis patients operated on in early childhood compared to controls. The goal was to find objective measurements that would correlate with the patient’s subjective self-evaluation of their own cosmetic appearance.

**Methods:**

The study population consisted of 49 patients from whom 41 had premature fusion of the sagittal and in 8 of metopic suture. There were 65 age and gender matching controls from The Finish National Register. The 3D photogrammetric models were created from all patients and controls. The images were analysed using Rapidform 2006. Facial landmarks were set by the standard Farkas points. Facial symmetry parameters were calculated by using the landmarks and the mirror shell of the face. Aesthetic evaluation was done from standard photographs using panels. Subjective satisfaction with one’s own appearance was evaluated using questionnaires.

**Results:**

Patients had the greatest asymmetry in the forehead area when compared to controls (symmetry percentage 59% versus 66%, *p* = 0.013). In the control group, the gap between the eyes was smaller than in the case group, resulting in an absolute 2 mm difference (*p* = 0.003). The area of the chin and the landmarks were more located on the left side in the patient group, resulting in up to a 1.1 mm difference between the groups (*p* = 0.003). Only a weak association was found between craniofacial symmetry and appearance evaluations.

**Conclusion:**

Patients operated on because of sagittal and metopic synostoses were found to have facial asymmetry at long follow-up. However, the differences were < 3 mm and not clinically important. The long-term aesthetical outcome of the surgery performed because of sagittal or metopic craniosynostosis based on the 3D image evaluation was good.

## Introduction

Craniosynostosis is defined as a condition where one or more sutures are prematurely fused. It is the second most common congenital cause of infant deformity occurring in 1 in 2000 live births [1, 2]. The premature fusion of the skull and facial bones may lead to changes in the normal function of the brain [3] as well as aesthetical disturbances, malocclusion [4, 5], and psychological disorders [6, 7]. Abnormal head shape may develop depending on which sutures are prematurely fused, the order in which they ossify, and the timing at which it happens. The diagnosis is made by clinical examination along with additional imaging methods like computer tomography (CT) and magnetic resonance imaging (MRI) [3, 8].

An average human face is not fully symmetrical, and the amount of facial asymmetry in normal population varies depending on the study method used [9]. Facial symmetry can be assessed by various methods, e.g., clinical evaluation, photography, cephalography, and three-dimensional (3D) imaging [9, 10]. There are numerous things that make the human face attractive and appealing depending on the culture and the environment [11]. However, meta-analyses indicate that averageness, symmetry, and sexual dimorphism are all crucial parts of attractiveness in both male and female faces and across culture [11].

3D surface imaging techniques have developed during the last years. There are different systems used for this purpose, including stereo-photogrammetry, laser scanning, structured light devices, and video-imaging. 3D photogrammetric imaging can be used for evaluation of any craniofacial deformity [12]. The accuracy of 3D photography is high, and the method is rapid, easy to apply, non-invasive, and reliable [1, 10, 13].

Most facial symmetry analyses on craniosynostosis patients have been done on unilateral plagiocephaly. In these studies, the facial asymmetry has been most prominent in the mid and lower part of the face, especially in the orbital and nasal area [14, 15]. It is known that restricted growth of midface and maxilla may lead to cross-bite and, consequently, facial asymmetry in the lower part of the face [16]. Nasolabial asymmetry is typically associated in cleft lip and palate patients [17, 18]. Often these patients have been associated with having a less pleasing aesthetical appearance [19].

Few studies have investigated how metopic or sagittal craniosynostosis affects the aesthetics in adults.

The aim of this prospective case–control study was to investigate the facial characteristics and facial aesthetics of the patients operated on during early childhood due to sagittal or metopic craniosynostoses, compared to controls by using 3D imaging. This study intends to research objective measurements that would correlate with the patient’s subjective self-evaluation of their own cosmetic appearance.

## Materials and methods

The basic patient cohort of this study consists of all patients with craniosynostoses who were treated in the Oulu University Hospital since 1977. Patients who were 18 years and older by December 2015 and had isolated non-syndromic craniosynostosis were invited to participate in the study. A total of 61 patients agreed to participate. Data on long-term follow-up of the patients treated because of sagittal suture synostosis and a description of the study protocol was published earlier [20]. Patients (*N* = 12) who at the study visit appeared to have syndromic craniosynostosis (3), ventriculoperitoneal shunt (1), or plagiocephaly (8) were excluded from this particular study (12). The final study group comprised of 49 patients (32 males, 17 females); 41 of them were operated on because of scaphocephaly and 8 for trigonocephaly. None of the patients had both scaphocephaly and trigonocephaly.

The control group consisted of age- and gender-matched persons, randomly chosen from a governmental database of the Finnish State Register. To provide a larger normal cohort, all controls were included in the analyses. From these 74 persons, nine had to be excluded due to extensive facial hair (etc. beard, moustache). As a result, 65 controls (33 males, 32 females) were included in the study. In the lower face and chin measurements, we used 61 controls, excluding four controls due to facial hair in the chin area.

### Processing and landmarking facial 3D models

**Fig. 1:**
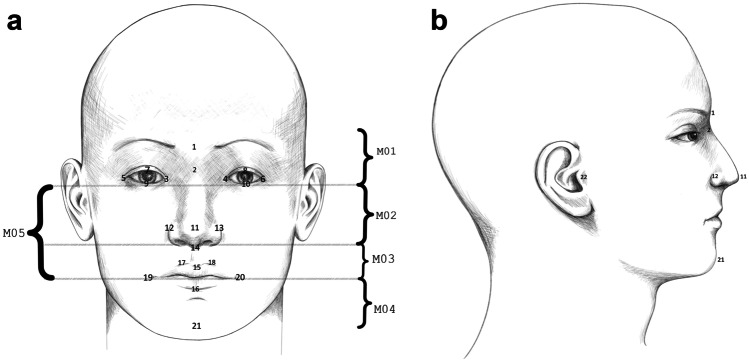
Areas of the face and the landmarks in front (**a**) and side (**b**) profile

The 3D images were taken using the 3dDMhead™ System (3dMD, Atlanta, GA, USA). The 3D images were processed and analysed using Rapidform 2006 (INUS Technology, Seoul, Korea). To prevent hair-induced disturbances, a tight nylon sock cap was fitted on each subject’s head prior to 3D imaging. All the 3D images were marked with the standard 21 Farkas landmark points (Table 1 and Fig. 1) [21]. In this study, we used additionally one landmark for the ears: tragion.

**Table 1:** Landmarks used for the analysis: Farkas 21-point landmarks and additional ear landmark. R = right, L = Left

Landmark	Abbreviation	Definition
1. Glabella	g	The most prominent midpoint between the eyebrows
2. Nasion	n	Midline point between the nasal root and nasofrontal suture, above the line that connects the 2 inner canthi
3,4. Endocanthion R/L	en	The point at the inner commissure of the eye fissure
5,6. Exocanthion	ex	The point at the outer commissure of the eye fissure
7,8. Pulpabrale superius	ps	The highest point in the mid-portion of the free margin of each upper eyelid
9,10. Pulpabrale inferius	pi	The lowest point in the mid-portion of the free margin of each upper eyelid
11. Pronasale	prn	The most protruded point of apex nasi
12,13. Alare R/L	al	The most lateral point on each alar contour
14. Subnasale	sn	The midpoint of the angle at the columella base where the lower border of the nasal septum and the surface of the upper lip meet
15. Labiale superus	ls	The midpoint of the upper vermilion line
16. Labiale inferius	li	The midpoint of the lower vermilion line
17,18. Christa philtri	cph	The point on each elevated margin of the philtrum above the vermilion line
19,20. Cheilion	ch	The point at each labial commissure
21. Pogonion	pg	The most prominent midpoint of the chin
22. Tragion R/L	tr	The point in the depth of the notch just above the tragus of the ear

All landmarks were placed on the images by the first author. After that, all possible distinct parts (like hair and ears) were removed from each image, in order to achieve the widest possible area of the face without disturbances for the analysis. Then, the position of the facial models was standardised using the previously described method [22].

### Facial symmetry parameters

For measuring symmetry parameters, the facial surface was mirrored across the *XY* plane (mid-sagittal plane). The face was divided into five different regions (Fig. 1). Region 1 — forehead (part of the face above mid-eye line), region 2 — eyes to nose (part of the face between subnasale (sn) and the mid-eye line), region 3 — nose to lips (part of the face between the mid-lip line (chL-chR) and subnasale), region 4 — chin (part of the face below the mid-lip line), and region 5 — eyes to lips (part of the face between the mid-lip line (chL-chR) and the mid-eye line). In addition, the analysis was done on the whole face as well. The average distance (in mm) between the original and the mirrored face was calculated for the whole face and separately for all the five facial areas. Additionally, the symmetry percentage (SP) was set to be the proportion of the facial areas where the distance between the original and the mirrored surface did not exceed 0.5 mm.

Landmark-based symmetry parameters were calculated by measuring the distance (in millimetres, mm) between midline landmarks or midpoint of landmark pairs and the sagittal plane. Additionally, we calculated the distances (in mm) between Endocanthion and Exocanthion landmarks on both sides for the case–control comparison of the distance between the eyes.

### Evaluation of aesthetics

All patients and controls were asked to express their satisfaction with their appearance in general and particularity facial appearance. The 100-mm Visual Analog Scale (VAS) was used a response of 100 mm corresponding to a “very satisfied” answer. This method and the results for scaphocephaly patients only were published previously [20].

Two different panels, the panel consisting of a dentist specialised in orthodontics and orthognathic surgery and layman panel, evaluated all cases by their facial appearance from usual photographs. The method and results for scaphocephaly patients were described previously [20]. The aesthetic outcomes were determined using a 100-mm VAS scale with 0 mm as the least attractive and 100 mm being the most attractive.

### Ethical board approval

The study was performed according to the principles of the Helsinki declaration. This is a part of a larger study that was approved by the Ethics Review Committee of the Northern Ostrobothnia Hospital District (No. 86/2013). Written informed consent was obtained from all participants.

### Statistical analyses

All statistical analyses were performed using the IBM SPSS software version 27 (SPSS Inc., Chicago, Illinois, USA). Two-tail *P* values of 0.05 or less were considered statistically significant. To assess normality, the Shapiro–Wilk normality test was used. Depending on the normality of the variables, the independent samples *T*-test or Mann–Whitney *U* test was used. The chi-squared test was used for nominal variables. Correlations between two variables were evaluated by the Spearman’s correlation.

## Results

The results of the 3D measurements were compared between the patient and the control group.

### Surface-based facial symmetry and landmark-based 3D evaluation

The degree of asymmetry in the forehead area was evaluated by the median symmetry percentage (SP) which was in the control group about 66% and patients 59% (*p* = 0.013) (Table 2). Correspondingly, the average distance (AD) between the original and the mirrored face was higher in the patients compared to the controls (*p* = 0.003) (Table 2). Patients had the median average distance of 0.51 mm, whereas controls had 0.46 mm. The remaining cases had no decreased symmetry in other facial areas. There was no statistically significant difference in the other facial areas considering symmetry measurements.

**Table 2:** Symmetry in the different regions of the face

**Area**	**Patients**				**Controls**				***p*-value**
	**Mean (SD)**	**95% CI**	**Median**	**IQR**	**Mean (SD)**	**95% CI**	**Median**	**IQR**	
**Whole face AD (mm)**	0.71 (0.22)	0.64–0.77	0.66	0.54–0.89	0.7 (0.23)	0.64–0.76	0.63	0.54–0.80	0.74^b^
**Whole face SP**	51.31 (10.33)	48.21–54.42	51.59		53.51(9.56)	51.15–55.88	54.58		0.25^a^
**Forehead AD (mm)**	0.55 (0.16)	0.51–0.60	0.51	0.44–0.61	0.48 (0.14)	0.44–0–51	0.46	0.37–0.56	0.003^b^*
**Forehead, SP**	57.96 (11.63)	54.62–61–31	58.78	49.97–65.62	63.95 (13.06)	60.71–67–19	65.65	55.17–72.37	0.013^b^*
**Chin AD (mm)**	1.09 (0.58)	0.92–1.27	0.93	0.69–1.46	1.19 (0.68)	1.02–1.36	1.07	0.68–1–53	0.61^b^
**Chin, SP**	34.68 (17.79)	29.27–40.08	33.67	18.14–44–60	33.20 (19.12)	28.46–37.94	30.99	16.47–46.91	0.61^b^
**Eyes to nose AD (mm)**	0.59 (0.23)	0.52–0.66	0.53	0.44–0.69	0.60 (0.23)	0.54–0.65	0.54	0.43–0.94	0.94^b^
**Eyes to nose, SP**	58.18(14.34)	54.06–62.31	58.00		57.38 (14.95)	53.68–61.09	58.55	44.12–62.75	0.77^a^

*AD* average distance; *SP* symmetry percentage; *SD* standard deviation; *IQR* interquartile range, lower and upper quartile; *95% CI* 95% confidence interval

^*^p-value < 0.05

^a^Independent samples *T*-test used

^b^Mann-Whitney *U* test used

In the patient group, the distance between the endocanthion points was larger than in the control group (36 mm and 34 mm, *p* = 0.003) (Table 3). There was no statistically significant (*p* = 0.30) difference between the exocanthion points in this study. The midpoint of the left and right tragion was found to be 0.23 mm shifted to the left from the mid-sagittal plane in the patients and 0.13 mm shifted to the right in the control group (Table 3).

**Table 3:** Landmarks and their distances to mid-sagittal plane (mm) and midpoint distances between landmarks (mm). Values were positive on the left and negative on the right side. The independent samples *T*-test was used

Landmark	Patients			Controls			*p*-value
	**Mean (SD)**	**95% CI**	**Median**	**Mean (SD)**	**95% CI**	**Median**	
**mid-En**	0.14 (0.64)	−0.04–0.33	0.15	0.20 (0.55)	0.064–0.34	0.085	0.61
**mid-Ex**	0.12 (0.69)	−0.08–0.32	0.13	0.32 (0.65)	0.15–0.48	0.26	0.13
**En-dist**	36.07 (3.18)	35.15–36.98	35.82	34.37 (2.75)	33.69–35.06	34.39	0.003*
**En-dist in Scaphocephaly**	36.46 (3.21)	35.45–37.48	36.36				0.001*
**En-dist in Trigonocephaly**	34.03 (2.25)	32.15–35.91	34.82				0.74
**Ex-dist**	88.92 (4.37)	87.66–90.18	88.05	88.05 (4.36)	86.97–89.13	87.87	0.30
**mid-tr**	−0.23 (0.85)	−0.48–0.02	−0.27	0.13 (0.89)	−0.09–0.35	0.11	0.032*
**pg**	0.41 (0.41)	−0.09–0.91	0.39	−0.70 (2.08)	−1.22 to −0.19	−0.50	0.002*
**li**	0.92 (1.57)	0.45–1.39	0.95	0.03 (1.51)	−0.35–0.40	−0.18	0.003 *
**ls**	0.52 (1.33)	0.12–0.92	0.82	−0.32 (1.29)	−0.64 to−0.00	−0.31	0.001*
**mid-cph**	0.17 (1.48)	−0.27–0.61	0.25	−0.53 (1.32)	−0.86 to−0.21	−0.45	0.012*

*SD* standard deviation, *95% CI* 95% confidence interval

^*^p-value < 0.05

In the chin area (MO4), controls tended to have deviation of the landmarks to the right side of the face, whereas in the patients to the left side (Table 3). Pogonion was 0.4 mm to the left in cases and 0.7 to the right in controls resulting in an absolute 1.1 mm difference (*p* = 0.003). Landmarks in the lip area (labiale superior, labiale inferior, and christa philtra) were dominant on the left (0.17–0.92 mm) in the patients and on the right in the control group (0.03–0.53 mm) (Table 3). Results that were statistically significant were not reported if the average distance (AD) difference between cases and controls was less than 0.01 mm, which was considered to fall into the range of measurement errors.

The distance between the tragion points along the *Z*-axis (sagittal line) appeared to be statistically significant (*p* = 0.03). Since it was only 0.36 mm mean, we assumed that noticing such a distance on the sagittal line is difficult and thus considered to have no clinical relevance.

### Correlations of 3D measurements with the subjective aesthetic evaluations

There was a weak correlation between the upper and the mid facial asymmetry and subjective self-evaluation of own appearance as well as panel evaluation of appearance (Table 4).

**Table 4:** Statistically significant correlations (*p* < 0.05) between facial symmetry and self-assessed satisfaction with appearance in general (Q1), facial aesthetics (Q2), and the panels’ VAS evaluation of appearance

Asymmetry parameter	Questions		Orthodontics panel	Layman panel
	Correlation coefficient (p-value)			
	*Q1*	*Q2*		
**Forehead AD**	−0.23 (0.016)*	−0,20 (0.03)*	−0.37 (< 0.001)*	−0.30 (0.002)*
**Forehead SP**	0.23 (0.016)*	0.23 (0.013)*	0.34 (0.001)*	0.28 (0.004)*
**Pg**			−0.25 (0.014)*	−0.36 (< 0.001)*
**Li**			−0.24 (0.017)*	−0.31 (0.002)*
**Ls**				−0.23 (0.02)*

^*^p-value < 0.05

## Discussion

This was a prospective case–control study conducted in order to evaluate the craniofacial characteristics, symmetry, and aesthetical outcome of patients operated on because of sagittal and metopic craniosynostosis. The main observation in this study was that the greatest asymmetry was found in the forehead area. Studies conducted on a healthy population show that facial symmetry is highest in the forehead area and respectively lowest in the mid-facial and chin area [23, 24]. In scaphocephaly and trigonocephaly patients, the premature ossification of the sutures, as well as the surgery itself, may have the greatest impact on the upper facial area. However, in plagiocephaly patients, nasomaxillary and mandibular asymmetry has been found [14, 15]. In our study, there was a minor difference in the average distance in the forehead area when compared to the controls. However, the symmetry percentage was significantly lower in the patient group which could be clinically relevant. Such findings have not been reported previously in scaphocephaly and trigonocephaly patients.

Metopic and sagittal craniosynostoses have been considered as “symmetrical” pathologies because of involvement only of the midline sutures [25]. To our knowledge, there are no studies dedicated to evaluation of symmetry in this patient group. When it comes to subjective facial attractiveness, fully symmetrical faces are not considered as appealing as mildly asymmetric ones [11]. The importance of beauty and the aesthetical perspective in this study is crucial because one of the treatment goals in craniosynostosis is a good outer appearance. Minor dis-satisfactions with one’s appearance may lead to psychological disorders and can affect daily life [6, 7].

The distance between the endocanthion points expressing the actual gap between the eyes was greater in the patient group, when compared to the controls. These results were analysed separately for scaphocephaly and trigonocephaly patients. The distance between endocanthions was found to be even wider when analysing the scaphocephaly patients separately (mean 36.46 mm). This was a statistically significant difference and of no clinical relevance due to only a 2-mm difference on average, when compared to the controls. Prominence of the forehead, so called frontal bossing, is one of the diagnostic criteria for scaphocephaly [2, 3, 25]. Whether the frontal bossing can cause a greater gap between the eyes is unclear. Mild hypertelorism can be produced by increased width of the cranial base [26]. However, hypertelorism by itself has not been mentioned in previous publications as a diagnostic criterion in scaphocephaly. In the trigonocephaly group, the distance between the endocanthions was approximately the same as in the control group. This is a positive result, since hypotelorism is a well-known preoperative finding in trigonocephaly patients [25].

Currently, there are still controversial findings considering which side of the face is larger in the average population. Some studies show that the left side is larger [27], whereas others report the right side to be larger [28, 29]. In addition, some studies show that while the left side of the face is larger in general, the chin is larger on the right side, and vice versa [30, 31]. This may be due to the developmental factors influence during the mandibular growth [31]. In our study, landmarks in the lower part of the face, such as pogonion, labiale superior/inferior, and christa philtra, were shifted to the right side in the control group. This suggests that in our study control population, the left part of the face was prominent.

Weak association was found between craniofacial symmetry and appearance evaluations. The symmetry of the forehead correlated with both, the self-evaluation and panels’ evaluation of facial aesthetics. This suggests that the eyes are typically the first part of the face that people pay attention to [32, 33]. The prominent and possibly atypical forehead shape can lead to a less pleasing aesthetical appearance. However, in the chin area, only the panels’ VAS evaluation correlated with the facial symmetry calculations. It is uncertain whether this weak correlation is suggestive of any clinical relevance. Do scaphocephaly and trigonocephaly patients pay less attention to the lower part of their own faces because the attention is biased towards more noticeable forehead asymmetry? Or does it simply play no role in their self-assessment since they may have gotten used to it? The absolute differences were small, and the correlation weak and thus these results must be evaluated with caution. However, it is pure speculation what could influence such a small difference in symmetry to correlate with the panels’ evaluation. Future research should focus on finding possible answers to these questions.

Scaphocephaly and trigonocephaly patients tend to have an abnormal shape of the forehead at long follow-up. This had an impact on the aesthetic results and the degree of symmetry in this facial area. All the differences found between the patients and control groups in this study were minimal, thus of no clinical significance. More studies are needed to determine whether the upper facial area is more asymmetrical in these patient groups and can it be considered as a typical residual feature after otherwise successful operative correction of these pathologies.

3D imaging is shown to be reliable and repeatable, even using landmark-based analysis [1, 13]. The accuracy varies, depending on the landmark, from 0.39 to 1.49 mm [34]. The geometric accuracy of the 3dDMhead™ System is < 0.2 mm, and reproducibility facial impression is reported to be 0.17 mm [35].

A strength of this study was that the patients and control groups were adjusted by age and sex of the case–control study design and the use of 3D imaging. 3D soft tissue imaging is a modern and non-ionizing tool for analysing facial symmetry [1, 36, 37]. The landmark-based analysis based on this imaging is feasible, reaching relatively high reproducibility according to the previous studies [38]. Additionally, several studies using computer tomography have shown that soft tissues have a remarkably good correlation with skeletal facial shape and facial asymmetry [39, 40], thus methods based on soft tissue analyses being valid for asymmetry analyses.

A limitation of the study is the subjective evaluation of one’s looks since it can be affected by age, culture, and modern trends which change over time. As the objective of the present study was to focus on facial soft-tissue characteristics, the shape of the skull will be a subject of future research.

## Conclusion

There was facial asymmetry found in the patients operated on because of sagittal and metopic synostoses at long follow-up, when compared to the controls. Sagittal synostosis patients tended to have greater distance between the eyes than controls and patients with metopic synostosis; however, the differences were < 3 mm and not clinically important. Results of 3D imaging should be taken with caution when evaluating facial symmetry. The long-term aesthetical outcome of the surgery performed due to sagittal or metopic craniosynostosis based on the 3D photogrammetric evaluation was good.

## Data Availability

This manuscript has no associated data.
